# A cohort description and comparison of four European national diabetes registries for the REDDIE project

**DOI:** 10.1111/dme.70112

**Published:** 2025-10-01

**Authors:** Jonah J. C. Thomas, Stefanie Lanzinger, Kathrine Kold Soerensen, Henrik Imberg, Stefanie Schmid, Emma Barron, Reinhard Holl, Ulrik Pedersen‐Bjergaard, Amanda Adler, Martina Vettoretti, Shivani Misra, Jonathan Valabhji, Kamlesh Khunti, Marcus Lind, Christian Torp‐Pedersen, Julia K. Mader, Pratik Choudhary, the REDDIE study group

**Affiliations:** ^1^ Diabetes Research Centre Leicester Diabetes Centre, Leicester General Hospital Leicester UK; ^2^ ZIBMT Institute of Epidemiology and Medical Biometry, Ulm University Ulm Germany; ^3^ German Center for Diabetes Research (DZD) Munich‐Neuherberg Germany; ^4^ Department of Cardiology North Zealand University Hospital Hillerød Denmark; ^5^ Department of Molecular and Clinical Medicine Institute of Medicine, Sahlgrenska Academy, University of Gothenburg Gothenburg Sweden; ^6^ Department of Metabolism, Digestion & Reproduction, Faculty of Medicine Imperial College London London UK; ^7^ Department of Diabetes and Endocrinology Chelsea and Westminster Hospital NHS Foundation Trust London UK; ^8^ Department Endocrinology and Nephrology Nordsjællands Hospital Hillerød Denmark; ^9^ Department Clinical Medicine University of Copenhagen Copenhagen Denmark; ^10^ Diabetes Trials Unit, Radcliffe Department of Medicine University of Oxford Oxford UK; ^11^ Department of Information Engineering University of Padova Padova Italy; ^12^ Department of Diabetes & Endocrinology Imperial College Healthcare NHS Trust London UK; ^13^ Department of Medicine Sahlgrenska University Hospital Gothenburg Sweden; ^14^ Department of Medicine NU‐Hospital Group Uddevalla Sweden; ^15^ Division of Endocrinology and Diabetology, Department of Internal Medicine Medical University of Graz Graz Austria

**Keywords:** diabetes, epidemiology, outcomes

## Abstract

**Aims:**

The Real‐world Evidence for Decisions in Diabetes (REDDIE) project aims to better understand how to use real‐world data (RWD) to advance research related to diabetes. To achieve this aim, four national registries (National Diabetes Audit (NDA (England)), Diabetes Patienten Verlaufsdokumentation (DPV (Germany)), Swedish National Registries (NDR), Danish National Registries (DNR)) are contributing data to the REDDIE project. This publication aims to describe the four registries and compare their unique strengths and limitations.

**Methods:**

Data regarding the four registries were extracted from their inception until 2024. Data regarding demographics, prescriptions, outcomes, lifestyle and diabetes‐specific variables (usage of continuous glucose monitors) were summarised.

**Results:**

A core set of variables was identified across all four registries in the REDDIE project. Demographic information, diabetes‐related medication, measures of glycaemic control and lifestyle factors are measured in all four registries. The DNR, NDA and NDR also contain wider prescription data, diagnosis data (cardiovascular disease, retinopathy) and mortality data. The DPV registry does not contain these data but contains detailed data on continuous glucose monitor and insulin pump usage. Differences in the methodologies employed and data fields collected were identified, including in data collection techniques, linkage processes, follow‐up protocols and the range of variables recorded. Even with this diversity in data collection, there remains a significant opportunity to perform collaborative analysis between the registries. By combining RWD collected in different populations and health care systems with diverse demographics, the transferability of evidence will increase, enabling research studies to be more representative and inclusive of diverse populations.

**Conclusion:**

The four registries that make up the REDDIE project contain many commonly collected variables. However, each registry presents specific strengths and limitations. By including all four databases, the REDDIE project benefits from the complementary strengths of each database.

**What’s new?**

- We compared the key characteristics of four national registries are contributing data to the REDDIE project.
- Many variables were consistently collected in all four registries however, each registry presents unique strength and limitations.
- It is important to understand these differences when interpreting the results of analysis conducted.

## 1 INTRODUCTION

The Real‐world Evidence for Decisions in Diabetes (REDDIE) project is a European Union‐funded project with the aim of understanding how to better use real‐world data (RWD) to advance research related to diabetes. Studies on RWD, including routinely collected information in large national registries, have received increasing attention and recognition in recent years.^1^, ^2^ While randomized controlled trials (RCTs), more specifically meta‐analyses of RCTs, are the accepted gold standard to investigate the efficacy and safety of new therapeutic agents, they have certain limitations. RCTs often include participants who meet strict inclusion and exclusion criteria and are followed up at regular intervals, but who often lack diversity in demographic characteristics, limiting representativeness. They are often younger, more compliant, and motivated, all of which limit the generalisability of results. Such studies often exclude the elderly and those with multiple long‐term conditions, children, those who are pregnant, and those with cognitive impairment. Further, even those in the control arm receiving standard of care are often seen more frequently and often receive a higher standard of care than that which is seen in routine clinical practice.^3^ In addition, once licensed, therapies are often used for a wider indication than was tested in the RCT. For example, drugs tested in those with high cardiovascular risk are then used in a larger population with lower cardiovascular risk.^4^ For this reason, the benefits and risks of a therapy assessed in an RCT may not reflect the reality of its use in wider clinical care.

Studies comparing treatment options using RWD may address some of these limitations and complement the results of RCTs as the data analysed collates information from varied sources, such as electronic health records from different health care settings, medical devices and wearables.^5^ They often include a more diverse, unrestricted population and reflect the wider clinical use of therapies in accordance with funding decisions and restrictions that may vary from country to country. However, when analysing RWD to determine treatment effectiveness, you must consider the presence of confounding factors accounted for by randomisation in an RCT. Disease registries are important sources of RWD since these data can provide real‐world evidence on the effectiveness and safety of therapeutic agents.^6^, ^7^

Over 33 million individuals live with diabetes in Europe, and this is expected to increase to 38 million by 2030.^8^ The REDDIE project aims to support the use of RWD to advance research related to diabetes and explores how RWD can complement the results of RCTs to improve evidence for the effectiveness and safety of innovative diabetes treatment (https://www.reddie‐diabetes.eu/). REDDIE also aims to improve the usage of RWD in diabetes for regulatory and payer decision‐making through the development of standards and guidance and a better understanding of the efficacy‐to‐effectiveness gap.

REDDIE will emulate previously conducted RCTs using RWD.^9^ This will allow comparison between the results observed from RCTs and the real world. To achieve this aim, we will analyse RWD from four population‐based registries: The Danish National Administrative Registers,^10^ the German Diabetes‐Patienten‐Verlaufsdokumentation (DPV),^11^ the Swedish National Diabetes Register (NDR)^12^ and the English National Diabetes Audit (NDA).^13^ Together, these databases provide broad population‐based coverage of the European population with diabetes and have been selected due to complementary strengths and limitations. We plan to analyse data separately within each registry, using shared statistical analysis plans, permitting us to observe similarities and differences in results between countries. Due to the differences between registries and challenges around data sharing between countries, harmonising variables across datasets was not deemed feasible. Instead, the results from analyses conducted in the four registries will be meta‐analysed to produce a summary of the findings. If a registry does not contain the necessary data to conduct an analysis (DPV lacks mortality data), it will not complete this analysis, and results from the registries with available data will be meta‐analysed.

This manuscript describes and compares data on characteristics of the populations within the four nationally representative databases and identifies strengths and limitations of each database in relation to the aims of the REDDIE project and wider diabetes research.

## 2 DESCRIPTION OF REGISTERS

### 2.1 The Danish National Registers (DNR)

In Denmark, a 10‐digit Civil Personal Register (CPR) number is given at birth or immigration, allowing for cross‐linkage of health and administrative registers at the individual level and enabling complete follow‐up. In 2024, the Danish population comprised 5,977,412 individuals. Every citizen is granted equal access to the health care system, including primary and hospital care. The Danish diabetes population is identified through data integration across multiple registers: (1) Danish Adult Diabetes Register^10^: Established in 2004, this register includes data from hospitals and general practitioners, covering over 70,000 adults with type 1 and type 2 diabetes. Among other things, it reports diagnosis year, BMI, smoking status, blood pressure, biomarkers and treatment processes. Data collection, initially manual, is now increasingly automated and reliable. (2) National Prescription Register^14^: Tracks all prescriptions dispensed since 1995, including dosage, dates and Anatomical Therapeutic Chemical (ATC) codes. (3) Danish National Patient Registry^15^: Contains records of hospital admissions and outpatient contacts since 1977, coded using the International Classification of Diseases (ICD‐9/10) and the Nordic Medico‐Statistical Committee Classification of Surgical Procedures. (4) Danish Cause of Death Registry^16^: Provides data on the date, cause and place of death from 1970 onwards. (5) The Danish Civil Registration System^17^: Includes information on the date of birth, sex, and civil and vital status. (6) Population Education Register^18^: Records the highest attained education level. (7) Income Statistics Registry^19^: Documents annual income data. (8) Laboratory Information System Databases^20^: Covers most blood tests from hospitals and general practitioners, with improved coverage starting in 2008 and all central laboratories being included since 2016. The DNR has produced publications on drug effectiveness and cardiovascular risk.^21^, ^22^, ^23^

### 2.2 Diabetes Patienten Verlaufsdokumentation, DPV—Germany, Austria, Luxembourg and Switzerland

The DPV was launched in 1995 in Germany and contains data from participating hospitals and practices specialising in diabetes care. The registry comprises data on around 760 000 children and adults with type 1 or type 2 diabetes as well as other forms of diabetes. Approximately 20% of adults with type 1 and 5% of adults with type 2 diabetes are included in DPV. The DPV registry can be regarded as representative for routine diabetes specialist care in Germany for adults with type 1 and type 2 diabetes. However, adult individuals with diabetes treated in primary care services are underrepresented.^24^ Contributing centres are located within Germany, Austria, Luxembourg and Switzerland. Demographic information, laboratory data (HbA1c), continuous glucose monitoring (CGM) and insulin pump data, documented medication, information on cardiovascular disease, and micro‐ and macrovascular complications are included. Twice a year, locally documented data are transmitted to Ulm University (Ulm, Germany) in pseudonymised form and in encrypted archives. DPV collects data from secondary care primarily, and people enter DPV either by manual entry by health care professionals or by automatic transfer from other hospital information systems. All data from local hospital information systems can be automatically transferred using the health‐level 7 interface, for example. Data are then aggregated into an anonymised, cumulative database. Benchmarking reports are created at Ulm University and circulated twice a year to all participating centres. In 2023, 707,035 people with diabetes (94,929 < 18 years, 612,106 ≥ 18 years) were documented in the DPV. The DPV registry is highly cited with many high‐impact publications, including comparisons with other databases.^25^, ^26^, ^27^

### 2.3 National Diabetes Audit (NDA)—England

The National Diabetes Audit (NDA) collects data on individuals living with, or at risk of developing, diabetes in England.^13^ The NDA has collated data on individuals living with diabetes registered with general practices in England since 2003, with almost complete general practice participation (>98%) in the last 5 years, making for almost total coverage of the diabetic population. In the latest data collection, covering January 2023 to March 2024, there were 272,400 individuals recorded with type 1 diabetes and 3,514,445 individuals recorded with type 2 diabetes. In 2017/2018, the NDA was extended to include individuals with a coded diagnosis of non‐diabetic hyperglycaemia (NDH, otherwise known as prediabetes). In the latest data collection, there were 3,746,940 individuals with a coded diagnosis of NDH. NDA data are collected from general practices in England by the General Practice Extraction System, which is an automated process, quarterly and collated centrally with data from secondary care services every 12 months using the National Health Service (NHS) number as a unique identifier. Demographic variables, as well as clinical care outcomes have been collected since the initiation of the NDA. Additional modules have been sequentially added, which utilise additional data from hospitals collected by clinical teams and provide data on pregnancy care (from 2012), foot care (from 2014) and insulin pump usage (from 2015). From 2017, specific drug therapies have been recorded (glucose‐lowering medications, antihypertensive medications and statins). Eye screening data have been included since 2019/2020. The NDA data can be linked by unique patient identifier (NHS number) to civil death registrations collated by the Office for National Statistics and Hospital Episode Statistics (HES), as well as a range of other national datasets and audits collected in England. Several key publications have been produced by NDA data.^28^, ^29^, ^30^

### 2.4 National Diabetes Register (NDR)—Sweden

The Swedish National Diabetes Register (NDR), established in 1996, collects detailed data on diabetes care, covering over 98% of children with T1D and approximately 90% of all individuals with diabetes in Sweden—equivalent to around 500,000 people annually.^31^ The 10% of missing coverage comes from health care providers not registering with the NDR or individuals opting out of their data being recorded. Data are gathered during routine clinical visits in primary and specialised care, using a combination of manual input by health care professionals and automated integration with electronic health records. The NDR records clinical measures such as HbA1c, BMI, blood pressure, lipids, creatinine levels, eGFR and markers for micro‐ and macroalbuminuria. It also includes information on smoking, physical activity, diabetes type, onset, treatments and complications like neuropathy and retinopathy. The register gradually became nationally representative of type 1 diabetes between 1998 and 2003, with increasing coverage over time. By 2009, it included over 90% of all primary care units and 70% of people.^32^ In 2018, Swediabkids, the paediatric diabetes register, was fully integrated into the NDR.^33^ CGM use began being recorded in the NDR in 2016,^34^ with specific CGM variables introduced in 2020.^35^ By linking the NDR to other Swedish national registers—such as the Cause of Death Register, the National Patient Register, the Prescribed Drug Register and the Longitudinal Integration Database for Health Insurance and Labour Market Studies (LISA)—more comprehensive data are obtained, including information on mortality, hospitalisations, dispensed medications (available since 2005), comorbidities and socioeconomic factors. Diagnoses and treatments are systematically coded using the ICD and ATC systems, and health interventions are coded using the Swedish Classification of Health Care Interventions (KVÅ). Several analyses have been produced from the NDR examining mortality, glycaemic control and heart failure.^36^, ^37^, ^38^

## 3 KEY VARIABLES IN THE REGISTRIES

**FIGURE 1:**
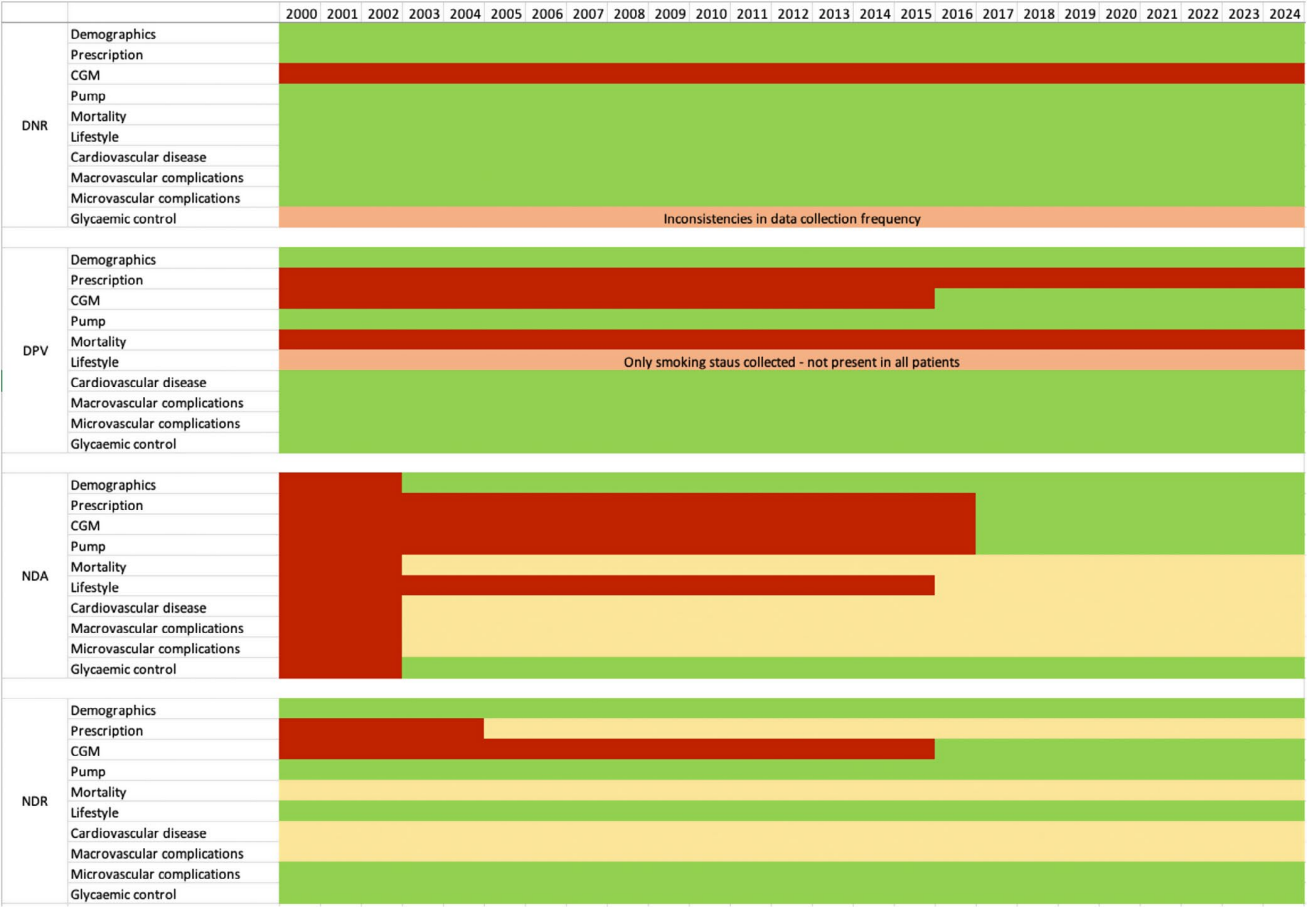
Timeline of data collection in Danish National Registers (DNR), Prospective Diabetes Follow‐up Registry (DPV), English National Diabetes Audit (NDA) and Swedish National Diabetes Register (NDR) since the year 2000. Red = not collected; yellow = available via linkage; orange = partially collected; green = fully collected.

Table 1 shows a list of key variables in each registry, including whether they are fully collected, partially collected (not collected in all individuals, may not be collected at regular intervals), available via linkage (is not available as part of the main registry but can be ascertained by linking with another registry) or not available. Table S1 presents information on data collection methodology. Figure 1 shows these key variables as a Gantt chart, illustrating how data availability in each registry has changed since the year 2000. The DNR has all variables available since 2000 except for information on usage of CGM. The English NDA started in 2003 and has collected prescription data since 2017/2018. Cardiovascular disease and data on other diagnoses, as well as mortality data, are available via linkage to other databases. The DPV database does not contain information on prescription or mortality but has collected data on diagnosis and glycaemic control, as well as physician‐documented medication. The Swedish NDR has collected data on glycaemic control and the use of CGM systems and insulin pumps, while mortality, diagnoses and prescription data can be accessed through linkage with other national registers.

**TABLE 1:** Key variables in each dataset, indicating whether they were collected, partially collected, available via linkage, or not available in the Danish National Registers (DNR), Prospective Diabetes Follow‐up Registry (DPV), English National Diabetes Audit (NDA) and Swedish National Diabetes Register (NDR).

Variable	DNR	DPV	NDA	NDR
*Demographics*				
Sex	Collected	Collected	Collected	Collected
Ethnicity	Collected	Partially collected	Collected	Partially collected (recorded as country of birth)
Socio‐economic status	Collected	Partially collected	Collected	Available through linkage
*Biochemistry*				
HbA1c	Partially collected	Collected	Collected	Collected
BMI	Partially collected	Collected	Collected	Collected
Blood pressure	Collected	Collected	Collected	Collected
Blood lipids	Collected	Collected	Collected	Collected
Creatinine	Collected	Collected	Collected	Collected
Micro−/macroalbuminuria	Partially collected	Collected	Collected	Collected
*Technology*				
Time in range (amount of time spent between 3.9 and 10 mmol/L (70 and 180 mg/dL) as measured by a CGM device)	Not available	Partially collected	Not available	Partially collected
Daily insulin dose	Not available	Collected	Not available	Available through linkage
Insulin pump usage	Collected	Collected	Collected	Collected
Hybrid closed loop usage	Not available	Collected	Collected	Collected
CGM usage	Not available	Collected	Collected	Collected
*Lifestyle*				
Smoking status	Partially collected	Collected	Collected	Collected
Physical activity	Not available	Collected	Not available	Collected
Nutrition	Not available	Not available	Not available	Not available
Alcohol consumption	Not available	Collected	Not available	Not available
*Diagnosis*				
Cardiovascular disease	Collected	Collected	Available through linkage	Available through linkage
Diabetic foot syndrome	Collected	Collected	Collected	Collected
Lower extremity amputations	Collected	Collected	Available through linkage	Available through linkage
Diabetic retinopathy	Collected	Collected	Available through linkage	Collected
*Prescription*				
Prescription data	Collected	Not available	Collected	Available through linkage
Anti‐diabetic medication	Collected	Collected	Collected	Available through linkage
Multiple daily insulin injection therapy	Not available	Collected	Partially collected	Available through linkage
*Outcomes*				
All‐cause mortality	Collected	Not available	Available through linkage	Available through linkage
Cause‐specific mortality	Collected	Not available	Available through linkage	Available through linkage

*Note*: Collected/green = collected at patient entry or mandatory collection at least every reporting cycle of that database, partially collected/orange = not mandatory and therefore less frequently collected. Red refers to “Not available” data while Yellow refers to data which is “Available through linkage”.

Abbreviations: BMI, body mass index; CGM, continuous glucose monitor; HbA1c, glycated haemoglobin; MDI, multiple daily injections.

**FIGURE 2:**
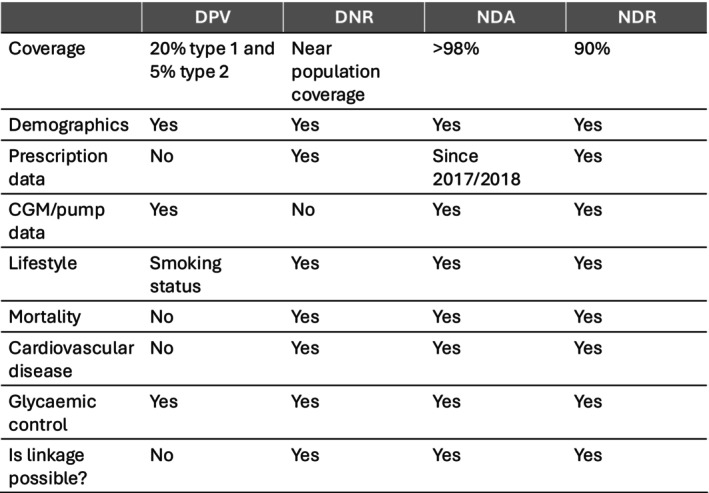
A comparison matrix table showing the similarities and differences between each registry involved in the REDDIE project. DNR, Danish National Registries; DPV, Prospective Diabetes Follow‐up registry; NDA, National Diabetes Audit; NDR, Swedish National Diabetes Register.

Figure 2 provides a summary of which variables are present across each of the REDDIE registries and which are unique to each.

## 4 DISCUSSION

Our study reveals substantial heterogeneity in the methodologies employed and data fields collected by national population‐based diabetes registries across four European countries, including differences in data collection techniques, linkage processes, follow‐up protocols and the range of variables recorded. Despite this diversity, a set of core shared variables that enhances comparability across datasets is possible to identify. These shared data demonstrate the potential for collaborative analyses, which may include meta‐analyses, validating and replicating studies using RWD over multiple registries, and federated learning analyses. Unifying RWD collected in different health care systems and from populations with diverse demographics will increase the strength of evidence derived from RWD and enable research studies to be more representative and inclusive of diverse populations.

### 4.1 Wider context of registry data usage

Each registry in the REDDIE consortium presents unique strengths and limitations. The NDA offers the largest number of people living with diabetes alongside comprehensive coverage of the national population affected by the condition. However, it is limited by having prescription data only since 2017/2018. The Swedish NDR covers nearly all individuals with type 1 and type 2 diabetes, providing comprehensive long‐term data on risk factors, complications and treatments. The DNR in combination allows collection of a diverse range of variables across the entire Danish population; however, data on diabetes‐specific variables are not as complete as the NDA. The DPV registry offers access to CGM data at a very high granularity compared with other registries; however, they lack prescription and mortality data. Both the DNR and the NDR provide national coverage. However, the Danish population has limited individuals of non‐white ethnic origin, but this reflects the Danish population rather than a bias in the registry. The NDA, DPV, NDR, and DNR were chosen to be part of the REDDIE project, as when analysed alongside each other, they allow a large range of research questions to be investigated while providing coverage across different health care systems in Europe.

### 4.2 Comparing registries

Previous work has reported the characteristics of nationally representative European diabetes registries.^39^ A systematic review by Bak and colleagues identified 12 clinical diabetes databases in Europe comprising over 7 million individuals living with diabetes. They found that nearly all registries measured body weight, HbA1c, serum lipid profiles and insulin treatment; however, reporting of other variables was inconsistent. This is similar to the datasets in the REDDIE project, with HbA1c and lipids being collected in all registries and prescription data in all except for the DPV. Lanzinger et al. previously identified paediatric diabetes registries globally.^40^ They compared seven databases and extracted information, including treatment, diabetes control, lifestyle factors and mortality. They again showed HbA1c, and lipid profiles were commonly collected alongside CGM usage, which is available in all REDDIE registries except the DNR.

### 4.3 Previous literature

The NDA, NDR and DNR collect data from primary and secondary care and facilitate linkage between the two and mortality data whilst linkage is not possible within DPV. This may influence the number of individuals living with diabetes who are recorded in each of the registries. Some individuals with diabetes may be solely managed from primary care and therefore complete population coverage may not be possible. The NDA utilises fully automated data collection in most modules with some exceptions (e.g. footcare, pregnancy). The DNR, DPV and NDR utilise a combination of automated and manual data entry. Automated data collection (not relying on a health care professional, or other individual, to enter data manually into the registry) allows greater consistency in how variables are collected and likely reduces the percentage of missing data. Further, automated data collection reduces data entry errors and time burden on health care professionals, and therefore to allow complete population coverage automated data collection becomes essential.^41^

### 4.4 Data collection and quality

All registries within REDDIE, except DPV, can perform linkage with other national registries or other data sources to allow more complex research questions to be explored. All registries except DPV use a unique person identifier to perform linkage across multiple national datasets. A range of variables can be linked, including socio‐economic status as well as mortality and diagnosis data. Linkage therefore allows a range of research questions to be posed that would not be possible with the data collected in each registry alone.^39^ Without linkage, many diabetes registers would not contain the required data to answer relevant research questions. Therefore, when setting up diabetes registers, it is important to consider the structure of other databases, which may contain useful data and ensure common variables are present in both data sources to allow future linkage.

### 4.5 Linkage to other registries

Registry data have previously been used to answer relevant diabetes‐related research questions for many years.^42^ In recent years, there has been an increasing number of initiatives which look to bring together registry data from multiple countries, the REDDIE project being one example. The DARWIN‐EU is a large collection of RWD databases in Europe.^43^ Currently, 20 data partners contribute to the network, allowing a range of research questions to be answered in a cross‐European sample. Further, CVD‐REAL is an initiative which brings together RWD databases from 13 countries across three continents.^44^ Such initiatives highlight that real‐world evidence is increasingly becoming a collaborative endeavour where ease of data access, while maintaining patient privacy, is crucial to advance research.

The four registries in REDDIE are not the only diabetes registries in Europe. Databases exist in, for example Scotland (Scottish national diabetes register), Finland (FinDM), the Netherlands (DPARD) and Latvia (Diabetes Register), which could contribute data to projects. Registries in other countries may allow the effect of different health care systems and drug reimbursement programmes to be assessed.

### 4.6 Strengths and limitations of registry data

There are many strengths associated with using registry data. Firstly, data are collected prospectively, allowing follow‐up to be performed on many people. Further, as no specific intervention is applied to participants, the effect of clinical, regulatory and policy decisions can be explored. For example, in the REDDIE project, the DNR, NDA and NDR will be used to investigate the effect of various drugs on cardiovascular outcomes in the real world without the artificial environment of a RCT. These three registries also offer national coverage of an entire population or for a specific disease group within a population. This approach provides large sample sizes for analysis as well as a near complete understanding of what is happening within the population. While RCTs exclude elderly, complex people and those from diverse ethnic backgrounds, RWD allows us to examine the effectiveness of therapies within these groups.^45^

Registry data does have several limitations. Firstly, due to strict privacy and data governance laws in both Europe and North America, accessing registry data can be challenging. These challenges can be reduced if researchers are in the same country as the registry data; however, extensive approval and ethical processes are required to ensure data is appropriately transferred, stored and analysed. Secondly, data quality in registries can vary greatly based on a wide range of factors, including how the data are entered into the databases. It is therefore important to understand how the data came to be in the registry and how this may impact the analysis conducted. There is also significant clinical heterogeneity in clinical decision making, which cannot always be fully captured. This means individuals may be prescribed different therapies based on their clinical characteristics, which can be hard to determine from the data captured in registries.

## 5 CONCLUSION

Each registry within the Real‐world Evidence for Decisions in Diabetes (REDDIE) project has unique strengths and limitations based on the variables available within them. By combining data and/or results from each of the four registries, each can showcase their respective strength, while any limitations can be covered by data from other sources.

## FUNDING INFORMATION

Funded by the European Union under contract number 101095556. Views and opinions expressed are however those of the author(s) only and do not necessarily reflect those of the European Union or European Health and Digital Executive Agency (HADEA). Neither the European Union nor the granting authority can be held responsible for them. This work has received funding from the UK Research and Innovation.

## CONFLICT OF INTEREST STATEMENT

KK has acted as a consultant, speaker or received grants for investigator‐initiated studies for Astra Zeneca, Bayer, Novo Nordisk, Sanofi‐Aventis, Servier, Lilly, Merck Sharp & Dohme, Boehringer Ingelheim, Oramed Pharmaceuticals, Pfizer, Roche, Daiichi‐Sankyo, Applied Therapeutics, Embecta and Nestle Health Science. KK is Chair of the NDA Research Steering Group. JKM is a member of advisory boards of Abbott Diabetes Care, Becton‐Dickinson, Biomea Fusion, Dexcom, Eli Lilly, Embecta, Medtronic, myLife, Novo Nordisk A/S, Pharmasens, Roche Diabetes Care, Sanofi‐Aventis, Tandem, Viatris and received speaker honoraria from A. Menarini Diagnostics, Abbott Diabetes Care, Dexcom, Eli Lilly, Medtrust, MSD, Novo Nordisk A/S, Roche Diabetes Care, Sanofi, Viatris and Ypsomed. She is a shareholder of decide Clinical Software GmbH and elyte Diagnostics and serves as CMO of elyte Diagnostics. AA has received travel support from Lilly related to a trial executive committee. ML has received research grants from Eli Lilly and Novonordisk and received honoraria or been a consultant for Boehringer Ingelheim, Eli Lilly, Nordicinfu Care, Novonordisk and Rubin Medical. JV was the National Clinical Director for Diabetes and Obesity at NHS England from April 2013 to September 2023. UPB is on the advisory panel of Novo Nordisk, Sanofi and Tandem; has received speaker fees from Abbott and Novo Nordisk; and has received research support from Novo Nordisk. PC has acted as a consultant, advisory board member and speaker for Medtronic, Novo Nordisk, Sanofi, Lilly, Abbott, Insulet, AstraZeneca, Dexcom, Glooko and Roche. He is an advisory board member for Ypsomed, Embecta and Vertex. He has received research support from Medtronic, Abbott, Novo Nordisk, Dexcom and Insulet.

## Supporting information


**Table S1.** Danish National Registers (DNR), Prospective Diabetes Follow‐up Registry (DPV), English National Diabetes Audit (NDA), and Swedish National Diabetes Register (NDR).
